# Supplementary material for: The Effect of Omega-3 Fatty Acid Supplementation on Serum Adipocytokines, Lipid Profile and Biochemical Markers of Inflammation in Recreational Runners
Source: Nutrients. 2021 Jan 29;13(2):456. doi: 10.3390/nu13020456 (PMC7912656; doi:10.3390/nu13020456)
Supplement: Supplementary file 1 [file nutrients-13-00456-s001.pdf]

dfMedica POLSKA

**NAME**		**COLLECTION DATE**	14/11/2018
**DOB**	--//--//--	**ACCEPTANCE DATE**	14/11/2018
**ID**		**RESULTDATE**	30/11/2018

Dried Blood Spot Fatty AcidProfile

Fatty Acid Group	Total	Reference Range
**Omega-3 Fatty Acids**	**6,82**	**4,32-11,54%**
Alpha-Linolenic (18:3n3)	0,24	0.05-1.50
Eicosapentaenoic (EPA, 20:5n3)	1,57	0.05-3.09
Docosapentaenoic-n3 (22:5n3)	1,10	0.03-2.71
Docosahexaenoic (DHA, 22:6n3)	3,91	0.58-5.00
**Omega-6 Fatty Acids**	**38,03**	**30,48-40,23%**
Linoleic (18:2n6)	25,95	7.25-27.87
Gamma-Linolenic (18:3n6)	0,40	0.09-0.37
Eicosadienoic (20:2n6)	0,25	0.21-0.47
Dihomo-γ-linolenic (20:3n6)	1,22	0.42-2.97
Arachidonic (AA, 20:4n6)	9,43	3.08-13.06
Docosatetraenoic (22:4n6)	0,62	0.06-4.54
Docosapentaenoic-n6 (22:5n6)	0,15	0.19-0.77
**Cis-Monounsaturated Fatty Acids**	**19,20**	**19.39-38.51%**
Palmitoleic (16:1n7)	0,62	0.01-3.83
Oleic (18:1n9)	16,69	13.87-32.81
Eicosenoic (20:1n9)	0,24	0.05-0.64
Nervonic (24:1n9)	1,65	1.05-7.12
**Saturated Fatty Acids**	**35,18**	**31.17-56.91%**
Myristic (14:0)	0,83	0.01-2.27
Palmitic (16:0)	21,44	11.27-31.75
Stearic (18:0)	11,88	8.56-17.28
Lignoceric (24:0)	1,03	0.81-4.39
**Trans Fatty Acids**	**0,66**	**0,0-2,33%**
Trans Palmitoleic (16:1n7t)	0,16	0.0-0.40
Trans Oleic (18:1t)	0,11	0.0-0.30
Trans Linoleic (18:2n6t)	0,39	0.0-0.40
SFA/MUFA	**1,83**	
Trans Fat Index	**0,50**	
Omega-3 Index	**6,60**	
AA/EPA	**6,03**	

Lab Director

ORDINE NAZIONALE DEI BIOLOGI ALBO PROFESSIONALE Dott. POLCARO PIERO N. 31481

Lab Director

SANNIO TECH the innovation cluster

MANAGEMENTSYSTEEM CERTIFICATIE DNV·GL ISO 9001

MANAGEMENTSYSTEEM CERTIFICATIE DNV·GL ISO 14001

Centro Diagnostico Delta s.r.l
Autorizzazione sanitaria prot. N°8449 del 30.11.2010 e s.m.i.
P.IVA 00269500625 82030 Apollosa BN

**NAME**		**COLLECTION DATE**	14/11/2018
**DOB**	--//--//--	**ACCEPTANCE DATE**	14/11/2018
**ID**		**RESULTDATE**	30/11/2018

Fatty Acid	Status	Result	Comments
SFA/MUFA	Optimal	**1,83**	From 1.7 to 2 = lowest risk sudden MI
Trans Fat Index	Optimal	**0,50**	From 0% to 2% = lowest risk sudden MI
Omega-3 Index	Intermediate Risk	**6,60**	> 8% = lowest risk sudden MI
AA/EPA	Suboptimal	**6,03**	From 1.5 to 3 = ideal range

SFA/MUFA

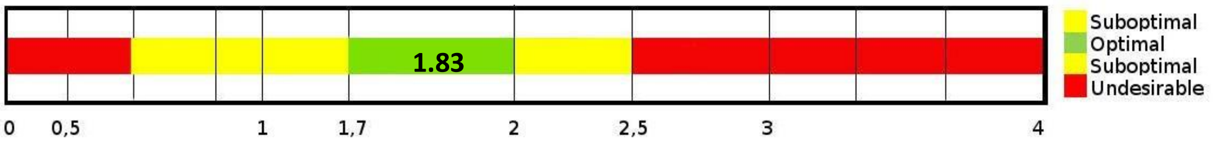

Trans Fat Index

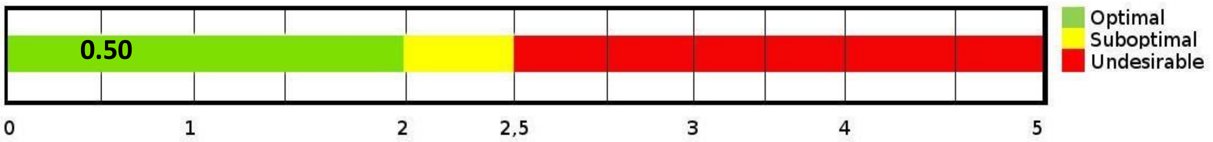

Omega-3 Index

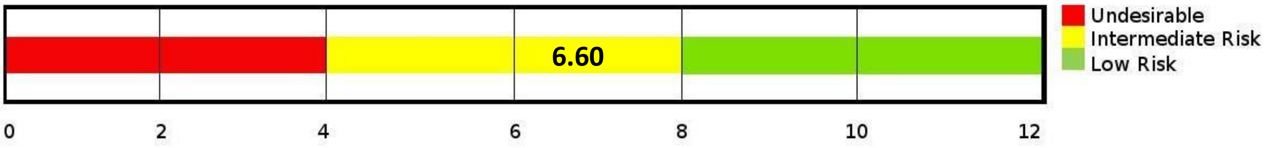

AA/EPA Ratio

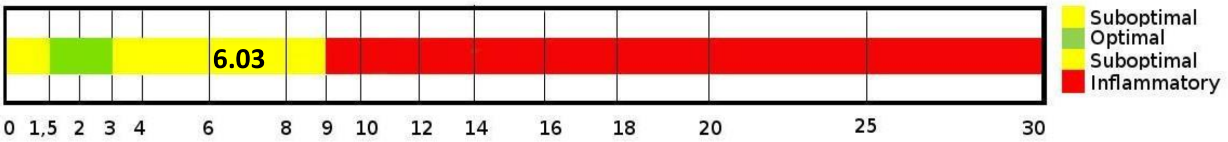

**NAME**		**COLLECTION DATE**	14/11/2018
**DOB**	--//--//--	**ACCEPTANCE DATE**	14/11/2018
**ID**	P18110014	**RESULTDATE**	30/11/2018

**SFA/MUFA**	**1.83**	0,5 - 1,7 and 2 - 2,5 =Suboptimal<br>1,7 - 2 =Optimal<br>0 - 0,5 and 2,5 - 4 = Undesirable
**Trans Fat Index**	**0,50**	2 - 2,5=Suboptimal<br>0 - 2=Optimal<br>2,5 - 5 = Undesirable
**Omega 3 Index**	**6.60**	0 - 4 =Undesirable<br>4 - 8 =Suboptimal<br>8 - 12 =Low Risk
**AA / EPA**	**6.03**	1,5 - 3 =Ideal Range
**Omega 6/Omega3**	**5.57**	3,5 - 5,5 = Idealrange

The reference ranges refer to healthy controls described in: Trattato di medicina di laboratorio – Diagnostica molecolare nella medicina di laboratorio IX Volume, Cap.19 Piccini, 2009.

The reference ranges for the Omega-3 index are described in: Harris SW Pharmacol.res.2007 and refer to people without cardiovascular disease.

Bibliography

Harris WS, von Schacky C: The Omega-3 Index: a new risk factor for death from coronary heart disease? Prev Med 2004, 39:212–220.

Lands B: A critique of paradoxes in current advice on dietary lipids.Prog Lipid Res 2008, 47:77–106.

Stark KD: The percentage of n-3 highly unsaturated fatty acids in total HUFA as a biomarker for omega-3 fatty acid status in tissues. Lipids 2008,43:45–53.

Armstrong JM, Metherel AH, Stark KD: Direct microwave transesterification of fingertip prick blood samples for fatty acid determinations. Lipids 2008, 43:187–196.

Bailey-Hall E, Nelson EB, Ryan AS: Validation of a rapid measure of blood PUFA levels in humans. Lipids 2008, 43:181–186.

Marangoni F, Colombo C, Galli C: A method for the direct evaluation of the fatty acid status in a drop of blood from a fingertip in humans:applicability to nutritional and epidemiological studies. Anal Biochem 2004, 326:267–272.

Marangoni F, Colombo C, Martiello A, Poli A, Paoletti R, Galli C: Levels of the n-3 fatty acid eicosapentaenoic acid in addition to those of alpha linolenic acid are significantly raised in blood lipids by the intake of four walnuts a day in humans. Nutr Metab Cardiovasc Dis 2007, 17:457–461.

Fratesi JA, Hogg RC, Young-Newton GS, Patterson AC, Charkhzarin P, Block TK, Sharratt MT, Stark KD: Direct quantitation of omega-3 fatty acid intake of Canadian residents of a long-term care facility. Appl Physiol Nutr Metab 2009, 34:1–9.

Metherel AH, Armstrong JM, Stark KD: Weekly changes in finger-tip prick blood highly unsaturated fatty acid (HUFA) composition with acute fish oil supplementation and washout in men and women. FASEB J2007, 21:A338–A339.

Rise P, Marangoni F, Martiello A, Colombo C, Manzoni C, Marconi C, Cattabeni F, Galli C: Fatty acid profiles of blood lipids in a population group in Tibet: correlations with diet and environmental conditions. Asia Pac J Clin Nutr 2008, 17:80–85.

Agostoni C, Galli C, Riva E, Colombo C, Giovannini M, Marangoni F: Reduced docosahexaenoic acid synthesis may contribute to growth restriction in infants born to mothers who smoke. JPediatr 2005, 147:854–856.

Agostoni C, Galli C, Riva E, Rise P, Colombo C, Giovannini M, Marangoni F: Whole blood fatty acid composition at birth: from the maternal compartment to the infant. Clin Nutr 2011, 30:503-505.

Magnusardottir AR, Skuladottir GV: Effects of storage time and added antioxidant on fatty acid composition of red blood cells at −20 degrees C. Lipids 2006, 41:401-404
